# Synthesis and Antimicrobial Activity of Novel Fluoroquinolone with Geranyl Amine Moiety

**DOI:** 10.3390/cimb48030260

**Published:** 2026-02-28

**Authors:** Ilmir R. Gilfanov, Svetlana A. Lisovskaya, Daria P. Gerasimova, Evgeniy S. Izmest’ev, Olga B. Babaeva, Denis V. Sudarikov, Pavel V. Gribkov, Iva I. Zadorina, Airat R. Kayumov, Liliya E. Nikitina

**Affiliations:** 1 Institute of Fundamental Medicine and Biology, Kazan Federal University, 420008 Kazan, Russia; ilmir.gilfanov@gmail.com (I.R.G.); s_lisovskaya@mail.ru (S.A.L.); iva.zadorina@yandex.ru (I.I.Z.); kairatr@yandex.ru (A.R.K.); 2 Faculty of Medicine and Biology, Kazan State Medical University, 420012 Kazan, Russia; 3 Scientific Research Institute of Epidemiology and Microbiology, 420015 Kazan, Russia; 4 Arbuzov Institute of Organic and Physical Chemistry, FRC Kazan Scientific Center of the Russian Academy of Sciences, 420088 Kazan, Russia; darya.p_gerasimova@mail.ru (D.P.G.);; 5 Institute of Chemistry, Federal Research Center “Komi Scientific Centre”, Ural Branch, Russian Academy of Sciences, 167000 Syktyvkar, Russia; evgeniyizmestev@rambler.ru (E.S.I.); pavelgribkov1999@icloud.com (P.V.G.); 6 Academy of Sciences of the Republic of Tatarstan, 420013 Kazan, Russia

**Keywords:** geranyl amine, fluoroquinolone, monoterpenoid, antimicrobial activity

## Abstract

The rapid emergence and global spread of antimicrobial resistance necessitate the development of novel antibacterial molecules. A promising strategy is the fusion of conventional drugs with fragments of natural compounds possessing various biological activity. In this study, we report the synthesis and antimicrobial activity of a novel fluoroquinolone carrying acyclic monoterpene moiety derived from geranyl amine. Compound **7** was obtained with a yield of 75% and characterized by NMR, HRMS, IR, UV, and single-crystal X-ray diffraction. The antimicrobial activity of the synthesized fluoroquinolone was assessed against MSSA and MRSA *S. aureus* clinical isolates, as well as *Candida* species and filamentous fungi. While exhibiting antibacterial activity lower than that of moxifloxacin against MSSA isolates (MIC 0.25–1 μg/mL), the compound demonstrated comparable or up to four-fold higher potency against MRSA isolates. The molecular docking confirmed the high binding affinity of compound **7** for DNA gyrase with the binding energy of −11.59 kcal/mol. In addition, moderate antifungal activity was observed against filamentous fungi (MIC 125–250 μg/mL). Thus, a novel fluoroquinolone represents a promising starting point for the design of antimicrobials for the treatment of staphylococcal infections complicated by fungal pathogens.

## 1. Introduction

The development and spread of antimicrobial resistance in microorganisms remain a serious global challenge, significantly limiting the therapeutic efficacy of conventional treatments and driving the search for new antimicrobial agents [1]. One rational and economically viable approach to addressing this issue is the hybridization of well-known drugs with functional moieties possessing various biological activities [2,3,4]. In this context, natural monoterpenoids, which are widely distributed in plant essential oils and exhibit antimicrobial and membrane-active properties, represent promising compounds for molecular hybridization and the development of novel agents with enhanced activity against resistant pathogens [5].

**Figure 1:**
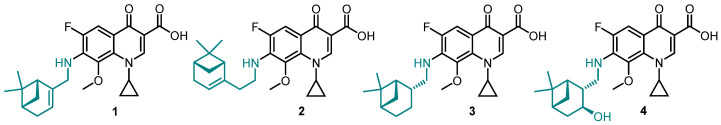
Structures of previously synthesized compounds **1**–**4**.

In our previous work, novel fluoroquinolones **1**–**4** carrying a bicyclic pinane moiety were synthesized (Figure 1) [6]. Among them, compound **4**, containing a *trans*-3-hydroxy-*cis*-myrtanylamine fragment, exhibited significant antibacterial activity with a minimum inhibitory concentration (MIC) of 0.015 µg/mL against *S. aureus* and was capable of penetrating the bacterial biofilm. In addition, compound **4** demonstrated antifungal activity with an MIC of 256 and 64 µg/mL against *C. albicans* and *A. niger*, respectively, whereas structurally related moxifloxacin, used as the reference drug, had no effect against the tested fungi. The studies on the mechanism of action of compound **4** indicated that the presence of both monoterpene and fluoroquinolone moieties contributes to the antimicrobial activity through a dual mechanism involving both the inhibition of topoisomerases by the fluoroquinolone core and membrane damage by pinane fragment [6].

These observations suggest that the conjugation of a monoterpene moiety with a fluoroquinolone core may represent a viable approach to modulate antimicrobial activity and overcome resistance. In contrast to previously reported compounds **1**–**4**, the introduction of an acyclic monoterpene fragment allows assessment of how conformational flexibility influences biological activity. Therefore, the present study aims to synthesize a novel fluoroquinolone carrying geranyl amine moiety and evaluate its antibacterial and antifungal activities.

For this purpose, geranyl amine obtained from geraniol was used. Geraniol is an acyclic monoterpene alcohol present in the essential oils of a number of plants, and it possesses a wide range of biological properties [5]. Its antioxidant, anti-inflammatory, antimicrobial, and antitumor activities have been reported previously [7]. Recently, we also synthesized a series of quaternary ammonium salts fused with various monoterpene fragments [8]. Among these compounds, the salt containing geraniol fragment exhibited high antimicrobial activity against several bacterial strains. Therefore, we assumed that the fusion of geranyl amine and fluoroquinolone fragments could lead to the formation of a new compound with unique biological properties.

## 2. Materials and Methods

### 2.1. General

All solvents were of reagent grade and used without further purification. Diisopropylethylamine (DIPEA) and acid **5** were purchased from Sigma-Aldrich (St. Louis, MO, USA). Geranyl amine **6** was prepared from geraniol (98% trans isomer, Sigma-Aldrich) according to the reported procedure [9]. Reaction progress and the purity of the obtained compounds were monitored by TLC on Sorbfil PTLC-AF-A-UF plates. Target compound **7** exhibited bright blue fluorescence under 365 nm UV light (eluent—EtOAc).

The IR spectrum was recorded on a Spectrum Two FT-IR spectrometer (PerkinElmer, Waltham, MA, USA). The melting point was determined on a Stuart SMP10 apparatus (Staffordshire, UK) and is uncorrected. The UV–Vis spectrum of a solution of the target compound (10 μg/mL) was recorded on a UV-2700 Spectrophotometer (Shimadzu, Kyoto, Japan).

The ^1^H and ^13^C NMR spectra were recorded on an Avance-II-500 spectrometer (Bruker, Bremen, Germany) at 500 and 125 MHz, respectively, in CDCl_3_ using the residual solvent signal as the internal reference. Chemical shifts (δ) are reported in ppm and coupling constants (*J*) in Hz. Complete signal assignment in the ^1^H and ^13^C NMR spectra was performed using two-dimensional homo-(^1^H-^1^H COSY) and heteronuclear (^1^H-^13^C HMBC, HSQC) experiments. The numbering of atoms used for signal assignment may differ from that recommended by IUPAC.

The X-ray diffraction (XRD) data of the single crystal were recorded on a Bruker D8 QUEST automated three-circle diffractometer (Bruker, Bremen, Germany) with a PHOTON III area detector and an I*μ*S DIAMOND microfocus X-ray tube (Incoatec, Geesthacht, Germany): λ (Mo *K*α) = 0.71073 Å, ω/ϕ scanning mode with a step of 0.5°. Data collection and indexing, determination, and refinement of unit cell parameters were carried out using the *APEX*4 software package (v2021.10–0, Bruker AXS, Madison, WI, USA). Numerical absorption correction based on the crystal shape, additional spherical absorption correction, and systematic error correction were performed using the *SADABS*-2016/2 software [10]. Using *OLEX*2 v1.3 [11], structures were solved by direct methods using the *SHELXT*-2018/2 program [12] and refined by full-matrix least-squares on *F*^2^ using the *SHELXL*-2019/1 program [13]. Nonhydrogen atoms were refined anisotropically. Positions of H(O/N) hydrogen atoms were determined from difference electron density maps and refined isotropically. The positions of hydrogen atoms of the methyl group were inserted using the rotation of the group with idealized bond angles; the remaining hydrogen atoms were refined using a riding model. Most calculations were performed using the *WinGX*-2021.3 software package [14].

The high-resolution mass spectrometry (HRMS) with electrospray ionization (ESI) was performed on an ESI-QTOF Impact II mass spectrometer with Elute UHPLC system (Bruker Daltonik GmbH, Bremen, Germany). The column YMC-Triart C18 (50 × 2.0 mm; 3 μm) was used. The column thermostat temperature was set at 40 °C and the autosampler temperature at 12 °C. Elution solvents Milli-Q water (A) + 0.1% formic acid and HPLC-grade acetonitrile + 0.1% formic acid (B) were used and elution gradient was the following: 0 min at 50% B, 2 min at 95% B, 4 min at 95% B, 4.1 min at 50% B, 6 min at 50% B with a flow rate of 0.3 mL/min. The injection volume was 2 μL. Measurements were made in positive mode in the range *m*/*z* 50–1500. The ESI source conditions were as follows: capillary voltage 4500 V, desolvation temperature 220 °C, drying gas nitrogen at a flow rate of 6 L/min. The samples were dissolved in HPLC-grade acetonitrile to a concentration of 0.02 mg/mL. The relative error in determining the exact mass values was no more than 5 ppm. The *m*/*z* values of the 100% peak in the ion cluster are given in the description. The Hystar (Bruker Daltonik GmbH, version 6.0), the OtofControl (Bruker Daltonik GmbH, version 5.2) programs were used to control the chromatograph and mass spectrometer. Data processing was performed by DataAnalysis software (Bruker Daltonik GmbH, version 5.3).

### 2.2. Synthesis of Compound ***7***

A solution of acid **5** (1.0823 g, 3.9 mmol, 1 equivalent) in DMSO (10 mL), containing geranyl amine **6** (1.2 g, 7.8 mmol, 2 equivalent) and DIPEA (1.36 mL, 7.8 mmol, 2 equivalent), was stirred at 70 °C for 8 h. On completion, the reaction mixture was diluted with 100 mL of water and extracted with EtOAc (3 × 20 mL). The combined organic layers were concentrated to a total volume of 20 mL. To this extract, 20 mL of water was added. The pH was then adjusted to 5–6 by adding 0.1 M HCl dropwise under vigorous shaking. The aqueous layer was discarded, and the organic layer was washed with water (2 × 20 mL). Next, a new portion of water (10 mL) was poured into the EtOAc extract. The pH was adjusted to 8–9 by the dropwise addition of 0.1 M NaOH with vigorous shaking. After discarding the aqueous layer, the organic one was washed with brine (2 × 20 mL). The resulting organic extract was diluted with 10 mL of water and then neutralized by several drops of 0.1 M HCl. The organic layer was separated, and the aqueous phase was extracted with EtOAc (2 × 20 mL). The combined organic extracts were dried over MgSO_4_, and the solvent was removed in vacuo. The solid residue was then crystallized from a mixture of acetone and water 1:1 by gradual solvent evaporation to produce crystals.

1-Cyclopropyl-7-((3,7-dimethylocta-2,6-dien-1-yl)amino)-6-fluoro-8-methoxy-4-oxo-1,4-dihydroquinoline-3-carboxylic acid (**7**). Yellow or orange crystals melting at 100 °C. Yield: 75%. IR, *v*, cm^−1^: 3338 (N–H), 3056, 2971, 2911 (C–H), 1711, 1619 (C=O), 1441 (C–F). UV, *λ* (A), nm (EtOH): 323 (0.426), 289 (1.817). NMR ^1^H (CDCl_3_) δ, ppm: 0.96–1.01 (m, 2H, H^11^), 1.15–1.21 (m, 2H, H^12^), 1.55 (s, 3H, H^10^`), 1.61 (s, 3H, H^9^`), 1.70 (s, 3H, H^4^`), 1.98–2.10 (m, 4H, H^5^`^,6^`), 3.72 (s, 3H, H^9^), 3.95–4.01 (m, 1H, H^10^), 4.06–4.12 (m, 2H, H^1^`), 4.48–4.53 (br.s, 1H, NH), 5.01 (t, 1H, *J* = 5.9, H^7^`), 5.32 (t, 1H, *J* = 6.7, H^2^`), 7.76 (d, 1H, *J* = 12.2, H^5^), 8.68 (s, 1H, H^2^). NMR ^13^C {^1^H} (CDCl_3_) δ, ppm: 9.62 (C^11,12^), 16.37 (C^4^`), 17.70 (C^10^`), 25.64 (C^9^`), 26.34 (C^6^`), 39.50 (C^10^), 39.74 (C^5^`), 43.32 (d, *J* = 7.3, C^1^`), 61.22 (C^9^), 107.32 (C^3^), 108.08 (d, *J*_F_ = 22.4, C^5^), 117.19 (d, *J*_F_ = 8.2, C^4a^), 121.13 (C^7^`), 123.70 (C^2^`), 131.80 (C^8a^), 133.27 (C^8^), 137.55 (d, *J* = 6.4, C^8^`), 137.66 (d, *J*_F_ = 12.3, C^7^), 140.43 (C^3^`), 149.44 (C^2^), 151.23 (d, *J*_F_ = 246.8, C^6^), 166.99 (C^13^), 176.86 (C^4^). ESI-HRMS, *m*/*z*: [M + H]^+^ calcd for C_24_H_30_FN_2_O_4_^+^ 429.2184, found 429.2186.

### 2.3. Antibacterial and Antifungal Activities

*Staphylococcus aureus* ATCC^®^ 29213™ and a series of clinical isolates (MSSA isolates 18, 25, 26, 27 and MRSA isolates 53, 65, 45, 67) provided by the Pharmaceutics Research Center of Kazan Federal University were used for antibacterial activity testing. Bacteria were maintained in LB broth (g/L: tryptone—10, yeast extract—5, NaCl—5, and pH of 7.0). A Mueller–Hinton broth (MH, BD Difco) was used in antibacterial activity tests.

Yeast strains (*Candida albicans* RKPG Y-4-01, *Candida tropicalis* RKPG Y-1513/784, *Candida parapsilosis* RKPG Y 1420/7) obtained from the Russian Collection of Pathogenic Fungi (P.N. Kashkin Research Institute of Medical Mycology, Saint Petersburg, Russia) were used to evaluate antifungal activity. The filamentous fungi (*Aspergillus niger* VKM-F-1119, *Fusarium oxysporum* VKM F-845, and *Alternaria tenuis* VKM F-1120) were obtained from the All-Russian Collection of Microorganisms (Pushchino, Russia). Fungi were cultivated in a liquid and on a dense Sabouraud nutrient medium. Sabouraud broth (for filamentous fungi) and RPMI 1640 (for yeast fungi) were used in antifungal activity tests.

Minimum inhibitory concentrations (MICs) of the compounds were determined using the broth microdilution approach in 96-well plates (SPL Life Sciences, Pocheon-si, Republic of Korea) in MH broth according to the EUCAST rules for antimicrobial susceptibility testing [15,16]. The bacterial and fungal suspensions (10^8^ and 10^6^ CFUs/mL, respectively) were subsequently diluted 1:300 with corresponding broth, and antimicrobials were added to final concentrations in the range of 0.0078–8 µg/mL for bacteria and of 1–1000 µg/mL for fungi. The MIC was determined as the lowest concentration of the compound for which no bacterial or fungal growth could be detected after 24 h of incubation. The MICs for mycelial fungi were assessed after 3–4 days of incubation. The median value obtained in four independent experiments was taken as the MIC of the compound.

### 2.4. Molecular Docking Analysis

Docking experiments were conducted using AutoDock 4.2.6 [17]. Prior to docking, the 2XCT structure was prepared by removing the S and U protein chains along with the associated DNA, ligands, and Mn^2+^ ions. The 5CDQ structure was similarly processed by deleting the R, S, T, and U chains, together with DNA, ligands, and Mg^2+^ ions. The 4URN structure was prepared by eliminating the B and C chains and the novobiocin molecules bound to them. All calculations were performed at pH 7.4 and 298 K, with the protein kept rigid while allowing full torsional flexibility of the ligands. Up to 50 conformers per ligand were generated using the Lamarckian genetic algorithm. Ligand geometries were optimized prior to docking at the B3LYP/6-31G(d,p) level of theory using ORCA [18,19]. Binding sites were defined based on the native co-crystallized ligands: ciprofloxacin and moxifloxacin within the DNA cleavage regions of 2XCT and 5CDQ, respectively, and novobiocin for 4URN. For 2XCT, the grid box was set to dimensions of 30 × 29 × 35 Å and centered at {1; 39; 61}. The same box size was applied to 5CDQ and 4URN, with grid centers at {50; −50; 61} and {33; 2.7; −3}, respectively. Visualization and figure preparation were carried out using PyMol 3.1.6.1 [20] and Chimera 1.19 [21].

## 3. Results and Discussion

### 3.1. Chemistry

**Figure 2:**
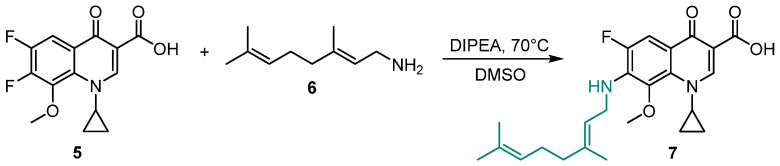
Synthesis of compound **7**.

The synthesis of compound **7** was carried out as shown in Figure 2 in accordance with the previously reported procedure [6]. The starting geranyl amine **6** was prepared as described earlier [9].

The reaction of starting acid **5** and geranyl amine **6** was performed at 70 °C for 8 h in the presence of diisopropylethylamine (DIPEA). A complex extraction procedure was applied for purification of the target compound. Next, fluoroquinolone **7** obtained with 75% yield was crystallized from a mixture of acetone and water 1:1 to afford crystals suitable for X-ray diffraction.

The structure of compound **7** was confirmed by spectroscopic methods (see Supplementary Materials). The ^1^H and ^13^C NMR spectra of the compound obtained display characteristic signals corresponding to both the monoterpene and fluoroquinolone fragments. In the HRMS experiment, the formation of mono- and dimeric species corresponding to protonated or sodium-adduct ions was observed. In addition, single crystal X-ray diffraction data were obtained for compound **7**. These crystallographic data, as well as IR and UV spectra, are available in the Supplementary Materials.

**Figure 3:**
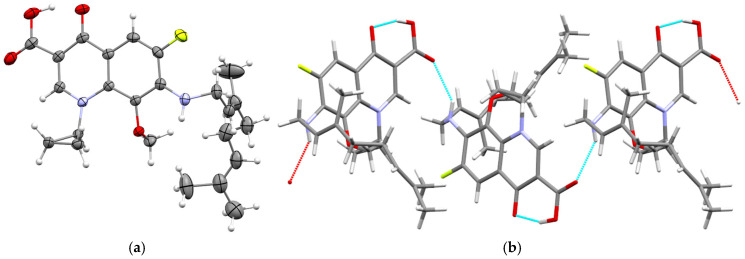
(**a**) Molecular structure of compound **7** in the crystal; (**b**) intra- and intermolecular interactions in crystal **7**. In X-ray diffraction figures, heteroatoms are always shown in different colors (for example, oxygen in red, sulfur in yellow).

Compound **7** crystallized in the space group *P*2_1_/*c*, and the asymmetric unit contains a single crystallographically independent molecule (Z′ = 1) (Figure 3a). In the molecule of compound **7**, an intramolecular O–H···O hydrogen bond is observed. The molecules are linked into a chain by N–H···O interactions (Figure 3b).

### 3.2. Antimicrobial Activity

The antimicrobial activity of fluoroquinolone **7** obtained in this study was evaluated against *S. aureus* since previously a similar molecule **4** containing a *trans*-3-hydroxy-*cis*-myrtanylamine fragment demonstrated superior activity compared to moxifloxacin [6]. Moxifloxacin, as a structurally related compound, served as a reference. The antimicrobial activity of the **7** was similar or slightly lower compared to moxifloxacin against MSSA typical strain and clinical isolates with MICs ranging from 0.25 to 1 μg/mL (Table 1). Fluoroquinolones are known for exhibiting bactericidal rather than bacteriostatic activity [22,23], while terpenes have lower bactericidal properties [5,7]; therefore, a bactericidal effect could be expected with high possibility from the side of fluoroquinolone moiety on bacteria. On the contrary, against MRSA isolates, **7** demonstrated the efficacy comparable to or exceeding that of moxifloxacin. The reasons for these effects are still speculative. MRSA has been reported to have altered cell wall peptidoglycan, teichoic acids, and lipids [24]. Apparently, the presence of terpene moiety impairs the diffusion of compound into these bacteria because of relatively high hydrophobicity, although low impact of hydrophobicity of compounds on their activity was demonstrated earlier [6].

In addition, the antifungal activity of compound **7** was evaluated against various fungi (Table 2), as the fusion of a pinane monoterpenoid with a fluoroquinolone scaffold in molecule **4** was previously shown to enhance antifungal activity [6]. Despite geraniol having been reported as a putative anti-*Candida* agent [25,26], compound **7** exhibited low activity against yeasts of the genus *Candida*, with an MIC of 500 μg/mL. In contrast, the compound showed moderate activity against filamentous fungi, including *Aspergillus*, *Fusarium*, and *Alternaria*. The MIC values for these species ranged from 125 to 250 μg/mL, significantly exceeding those of fluconazole, a drug widely used in clinical practice. Apparently, the presence of a highly hydrophobic terpene fragment induces the intracellular accumulation of reactive oxygen species, which in turn affects cell membrane permeability as reported previously for geraniol [27].

Particularly of note, while antifungal activity has been reported for some fluoroquinolones [28], the effect was observed at concentrations (1 mg/mL and higher), suggesting that compound **7** represses fungal growth, apparently due to the activity of the terpene moiety, thus exhibiting rather fungistatic features [27,29]. Nevertheless, fungicidal activity can be expected for **7** due to fluoroquinolone moiety at high concentrations, which are not relevant for clinical application.

Additionally, the terpene moiety may mimic the action of hydrophobins, thereby influencing fungal growth rate and sporulation. Hydrophobins are low-molecular hydrophobic proteins located on the surface of hyphae that form an amphipathic protein film and reduce the surface tension of water, thereby limiting rapid fungal growth [30]. Furthermore, hydrophobins are also present on the spore surface. Since the initial fungal inoculum contained mainly fungal spores, their interaction with **7** could inhibit the germination to a certain extent, or vice versa, diminish its antifungal activity, thus explaining the lower activity compared to reported data for geraniol [25,26]. From a practical standpoint, this effect is of interest for inhibiting the germination and rapid colonization of the human body by fast-growing mycelial fungi, challenging further modifications of the fusion to find the optimal chemotype suitable for the one-shot treatment of bacterial–fungal mixed infections.

### 3.3. Molecular Docking

To evaluate the *in silico* affinity of compound **7** for the bacterial targets in *S. aureus*, molecular docking simulations were performed against DNA gyrase and topoisomerase IV. The crystal structures employed were 2XCT (DNA gyrase with ciprofloxacin), 5CDQ (DNA gyrase with moxifloxacin), and 4URN (the N-terminal subunit *ParE* subunit of topoisomerase IV in complex with novobiocin) [31,32]. All structures were obtained from the RCSB Protein Data Bank “https://www.rcsb.org/ (accessed on 11 February 2025)”.

Previous structure–activity relationship studies on ciprofloxacin and norfloxacin analogs demonstrated that direct introduction of a bulky monoterpene substituent onto the piperazine ring led to a complete loss of antibacterial activity. In contrast, the incorporation of a flexible acetic or propionic acid linker between the quinolone core and the bulky moiety restored activity, albeit specifically against Gram-positive *S. aureus* [33]. Accordingly, we hypothesized that replacing the rigid monoterpene fragments with a linear and more flexible geranyl moiety would preserve antibacterial efficacy, as this modification is less likely to sterically hinder penetration into the DNA cleavage site of topoisomerases.

**Figure 4:**
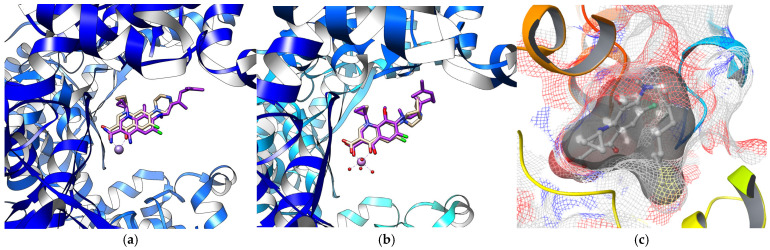
Predicted binding modes of compound **7** (violet) in comparison with (**a**) ciprofloxacin in 2XCT; (**b**) moxifloxacin in 5CDQ; and (**c**) shape complementarity of **7** withing the active site of 4URN.

The docking results (Table 3) confirmed this hypothesis. Simulations against the DNA gyrase structure 2XCT revealed high binding affinity of compound **7** for this enzyme, with the binding energy of −11.59 kcal/mol, compared to −12.83 kcal/mol for the redocked native ligand ciprofloxacin [33]. Notably, the binding mode of compound **7** closely resembles that of ciprofloxacin and features the same key interactions: chelation of Mn^2+^ ion by both carbonyl groups of the fluoroquinolone core, and a hydrogen bond between the carboxylic acid group and Ser1084 (Figure 4a).

Docking of compound **7** to moxifloxacin-bound DNA gyrase structure 5CDQ also yielded a binding energy comparable to that of the native ligand (ΔG = −12.75 kcal/mol vs. −13.07 kcal/mol for moxifloxacin). The spatial arrangement of compound **7** was identical to that observed with the 2XCT target, and the same molecular interactions were retained: coordination of the carbonyl and carboxyl groups with Mn^2+^ and Ser1084, respectively, along with an additional interaction of the carboxylic group with Arg122 (Table 3, Figure 4b).

The only available crystal structure of *S. aureus* topoisomerase IV (4URN) is complexed with novobiocin and lacks metal ions in the binding site. Since fluoroquinolone binding is critically dependent on metal coordination, high-affinity interactions with compound **7** were not expected. The docking data are consistent with this, yielding only modest binding energies, which can be further attributed to the limited number of polar contacts. Nevertheless, compound **7** displays excellent shape complementarity to the binding pocket (Figure 4c).

The lipophilicity of compound **7** was calculated using the SwissADME online tool “https://www.swissadme.ch/ (accessed on 11 February 2025)” and compared to that of moxifloxacin. Compound **7** exhibits a LogP value of 4.30, whereas moxifloxacin has a LogP of 1.54. The markedly higher lipophilicity of compound **7** is consistent with the incorporation of the terpene-derived geranylamine moiety. This pronounced hydrophobic character is expected to facilitate membrane penetration and may enhance accumulation within the lipid bilayer, potentially contributing to antibacterial activity through membrane-associated mechanisms.

## 4. Conclusions

In this work, a novel fluoroquinolone carrying a geranyl amine moiety was successfully synthesized. The compound demonstrated promising antimicrobial properties compared to moxifloxacin, although with similar or lower activity against MSSA but higher efficiency against MRSA clinical isolates. On the other hand, although moderate, the observed antifungal activity against filamentous fungi highlights the multifunctional potential of monoterpene-containing fluoroquinolone hybrids. Although the current compound does not outperform reference antifungal drugs, its activity range and structural features provide a valuable basis for further chemical modification.

Comparison of compound **7** with the previously reported fluoroquinolone **4** suggests that the nature of monoterpene fragments plays a critical role in determining antimicrobial potency. While the rigid bicyclic pinane moiety favored high activity against MSSA [6], the more flexible geranyl fragment reduced activity against susceptible strains but enhanced or maintained activity against MRSA isolates. This behavior may reflect differences in membrane interactions, supporting the hypothesis that monoterpene fragments can contribute to membrane-associated effects in addition to the inhibition of bacterial topoisomerases by the fluoroquinolone core.

Overall, these findings support the concept that conjugation of fluoroquinolones with monoterpene moieties is a promising strategy for development of new antimicrobials for the treatment of staphylococcal infections complicated by fungal pathogens.

## Figures and Tables

**Table 1:** Antibacterial activities of **7**.

Strains	MIC, µg/mL	
	7	Moxifloxacin
*S. aureus* ATCC29213	0.25	0.125
MSSA 18	0.25	0.125
MSSA 25	0.25	0.125
MSSA 26	1	0.06
MSSA 27	0.25	0.06
MRSA 30	1	4
MRSA 53	1	2
MRSA 65	2	2
MRSA 45	1	2
MRSA 67	0.5	2

**Table 2:** Antifungal activities of **7**.

Strains	MIC, µg/mL					
	7	Ketoconazole	Itraconazole	Fluconazole	Terbinafine	Moxifloxacin
*C. albicans*	500	7.8	15.6	15.6	62.5	>1000
*C. tropicalis*	500	7.8	15.6	62.5	62.5	>1000
*C. parapsilosis*	500	15.6	15.6	125	3.9	>1000
*A. niger*	125	15.6	15.6	500	3.9	>1000
*F. oxysporum*	125	15.6	15.6	500	7.8	>1000
*A. tenuis*	250	15.6	15.6	500	7.8	>1000

**Table 3:** Results of molecular docking of **7** to 2XCT, 5CDQ, and 4URN.

Entry	Calculated Docking Parameters for 7	*S. aureus* Target Type		
		2XCT	5CDQ	4URN
1	ΔG, kcal/mol	−11.59	−12.75	−8.86
2	LE ^[a]^, kcal/mol	−0.37	−0.41	−0.29
3	Intermolecular interactions	O=C–O^−^···HO(Ser1084) O^−^–C=O···Mn^2+^···O=C	O=C–O^−^···NH_2_(Arg122) O=C–O^−^···HO(Ser84) O^−^–C=O···Mg^2+^···O=C	O=C–O^−^···NH_2_(Arg138) CH_2_–NH···HO(Thr34)

^[a]^ Ligand efficiency (LE) is defined as ΔG/*n*, where *n* is the number of heavy atoms (non-hydrogen atoms) in the molecule.

## Data Availability

The following data is available from the corresponding author.

## Supplementary Materials

The following supporting information can be downloaded at: https://www.mdpi.com/article/10.3390/cimb48030260/s1, and consists of ^1^H and ^13^C NMR, HRMS, IR, and UV spectra. CCDC 2516320 contains the supplementary crystallographic data for this paper. These data can be obtained free of charge via https://www.ccdc.cam.ac.uk/structures/ (or from the CCDC, 12 Union Road, Cambridge CB2 1EZ, UK; Fax: +44 1223 336033; E-mail: deposit@ccdc.cam.ac.uk).
