# Supplementary material for: Synthesis and Antimicrobial Activity of Novel Fluoroquinolone with Geranyl Amine Moiety
Source: Curr Issues Mol Biol. 2026 Feb 28;48(3):260. doi: 10.3390/cimb48030260 (PMC13024941; doi:10.3390/cimb48030260)
Supplement: Supplementary file 1 [file cimb-48-00260-s001.zip › HRMS.pdf]

Compound Spectrum SmartFormula Report

Analysis Info

		Acquisition Date	3/13/2025 12:21:32 PM
Analysis Name	D:\Data\IOPC\Babaev\2025\Nikitina\GerNMoxi_28_1_1920 — blank CH3CN_25_1_1919.d		
Method	LCMS_pos_6min_50-95B_50_1900mz.m	Operator	Demo User
Sample Name	GerNMoxi	Instrument	impact II 1825265.00000
Comment	50mkl_MeOH<br>Original analysis: 'GerNMoxi_28_1_1920.d',<br>Subtracted analysis: 'blank CH3CN_25_1_1919.d' (Xpose, 0.5s, 5),<br>Processed with: 'DataAnalysis 5.3.556',<br>Date: 2025-03-13T12:30:00		

Acquisition Parameter

Source Type	ESI	Ion Polarity	Positive	Set Nebulizer	2.0 Bar
Focus	Not active	Set Capillary	4500 V	Set Dry Heater	220 °C
Scan Begin	50 m/z	Set End Plate Offset	-500 V	Set Dry Gas	6.0 l/min
Scan End	1900 m/z	Set Collision Cell RF	1500.0 Vpp	Set Divert Valve	Source

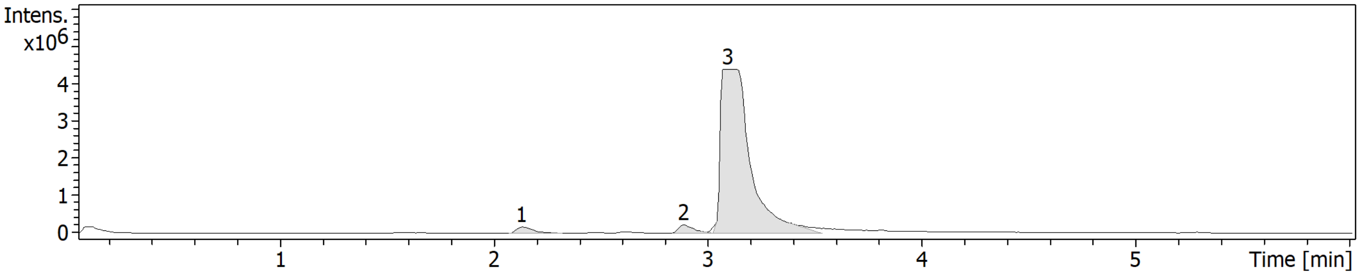

#	RT [min]	Int. Type	Trace	Area	Range [min]	S/N	I
1	2.1	Chromatogram	BPC +All MS	860543	2.1 - 2.3	100.9	166889
2	2.9	Chromatogram	BPC +All MS	1073914	2.8 - 3.0	131.0	219514
3	3.1	Chromatogram	BPC +All MS	42616212	3.0 - 3.5	2674.5	4381695

Cmpd 1, 2.1 min

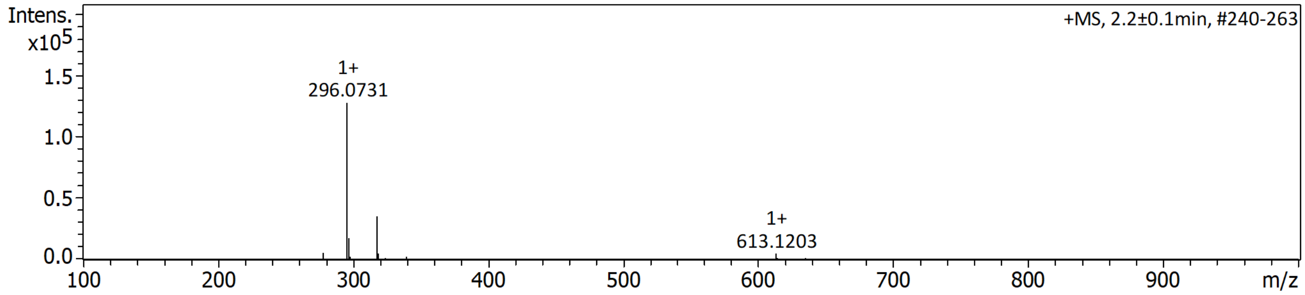

Cmpd 2, 2.9 min

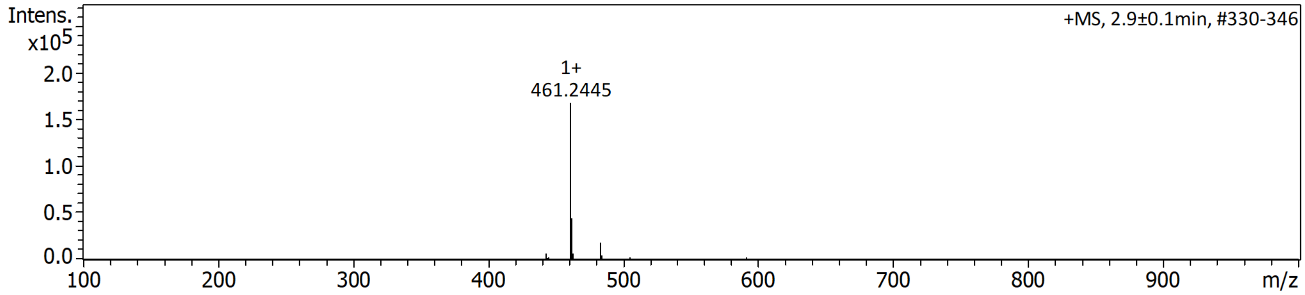

Compound Spectrum SmartFormula Report

Cmpd 3, 3.1 min

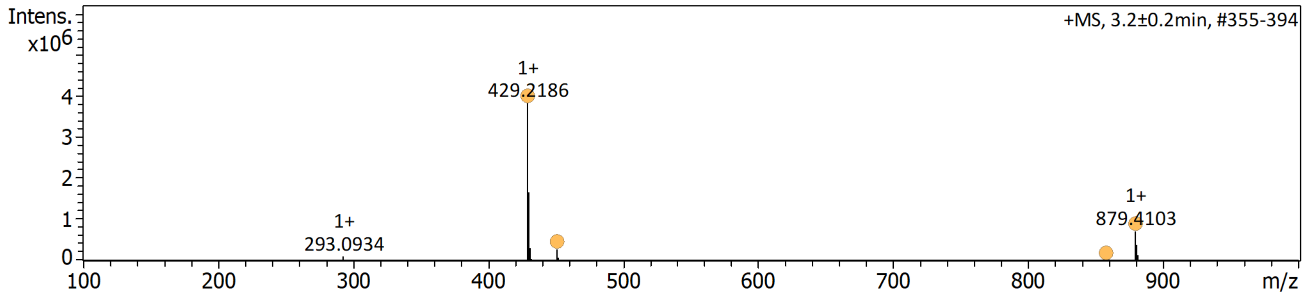

Meas. m/z	#	Ion Formula	m/z	err [ppm]	Mean err [ppm]	rdb	N-Rule	e¯ Conf	mSigma	Std I	Std Mean m/z	Std I VarNorm	Std m/z Diff	Std Comb Dev
429.218642	1	C24H30FN2O4	429.218412	-0.5	-1.3	11.0	ok	even	96.7	130.4	n.a.	n.a.	n.a.	n.a.
451.200172	1	C24H29FN2NaO4	451.200356	0.4	-1.6	11.0	ok	even	8.5	12.0	n.a.	n.a.	n.a.	n.a.
857.428263	1	C48H59F2N4O8	857.429548	1.5	1.8	21.0	ok	even	22.9	24.7	n.a.	n.a.	n.a.	n.a.
879.410280	1	C48H58F2N4NaO8	879.411492	1.4	0.5	21.0	ok	even	12.7	16.9	n.a.	n.a.	n.a.	n.a.
