# Supplementary material for: Synthesis and Antimicrobial Activity of Novel Fluoroquinolone with Geranyl Amine Moiety
Source: Curr Issues Mol Biol. 2026 Feb 28;48(3):260. doi: 10.3390/cimb48030260 (PMC13024941; doi:10.3390/cimb48030260)
Supplement: Supplementary file 1 [file cimb-48-00260-s001.zip › SI CIMB-manuscript.pdf]

Table S1. Crystallographic data and X-ray structural experiment parameters for the single crystal.

**Compound**	**7**
Empirical formula	$C_{24}H_{29}FN_2O_4$
Formula weight	428.49
Crystal system	monoclinic
Space group	$P2_1/c$ (No. 14)
Unit cell dimensions:<br>*a*, *b*, *c*, Å;<br>α, β, γ °	13.2222(13),<br>13.9846(13),<br>13.5854(13);<br>90,<br>118.074(3),<br>90
Volume, Å^3	2216.5(4)
Z and Z′	4 and 1
Calculated density, g cm^{-3}	1.284
Absorption coefficient, mm^{-1}	0.093
F(000)	912
Crystal size, mm^3	0.502 × 0.491 × 0.379
θ range for data collection, °	2.238 to 26.998
Index ranges	$-16 \leq h \leq 16$,<br>$-17 \leq k \leq 17$,<br>$-17 \leq l \leq 17$
Reflections collected	48297
Independent reflections	4833
R_{int}	0.0686
R_σ	0.0368
Observed Data [$I > 2\sigma(I)$]	4044
Completeness to θ = 25.242°, %	99.9
Max. and min. transmission	0.7460 and 0.5594
Data / restraints / parameters	4833 / 0 / 292
Goodness-of-fit on F^2	1.034
Final *R* indices [$I > 2\sigma(I)$]	$R1$ = 0.0450, $wR2$ = 0.1188
R indices (all data)	$R1$ = 0.0550, $wR2$ = 0.1257
Largest diff. peak and hole, *e* Å^{-3}	0.261 and –0.209
CCDC number	2516320

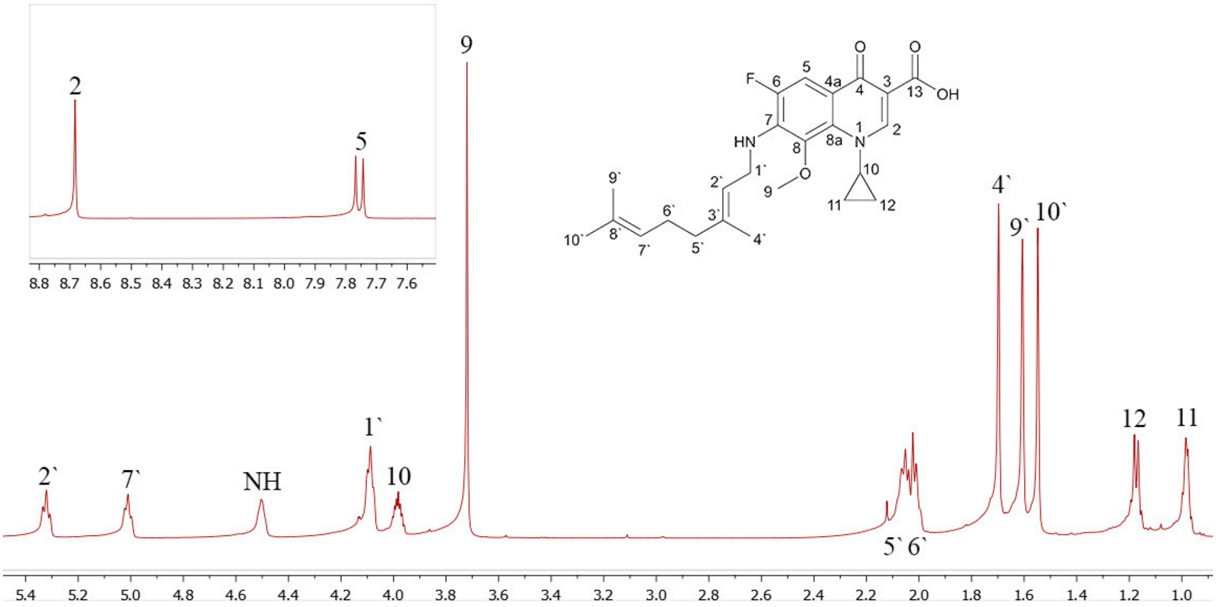

Figure S1. ^{1}H NMR spectrum of compound **7**.

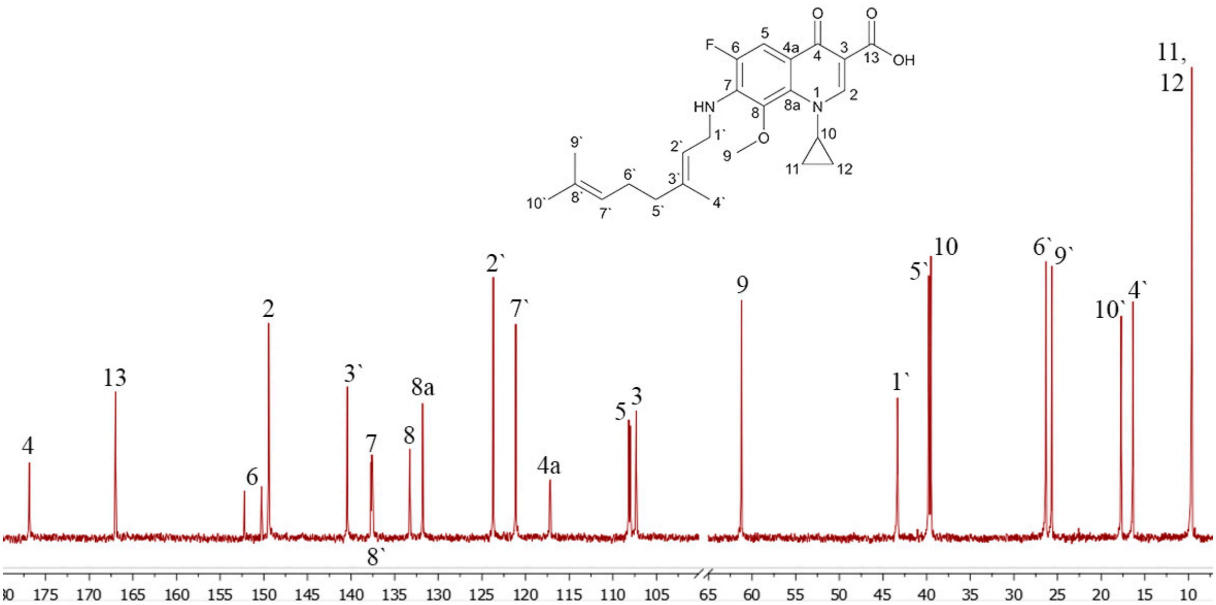

Figure S2. ^{13}C NMR spectrum of compound **7**.

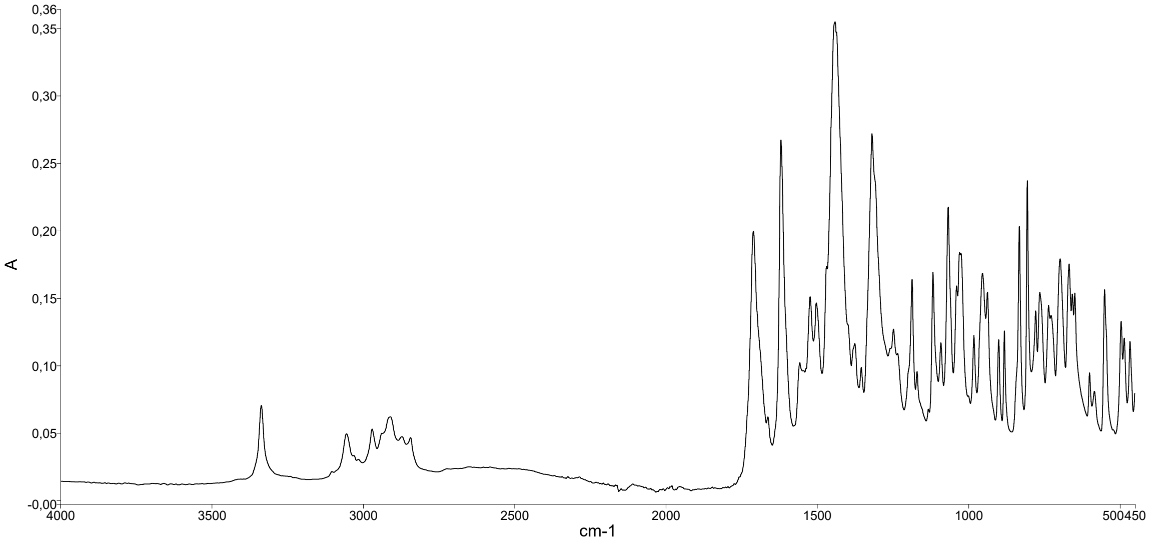

Figure S3. The IR spectrum of compound **7**.

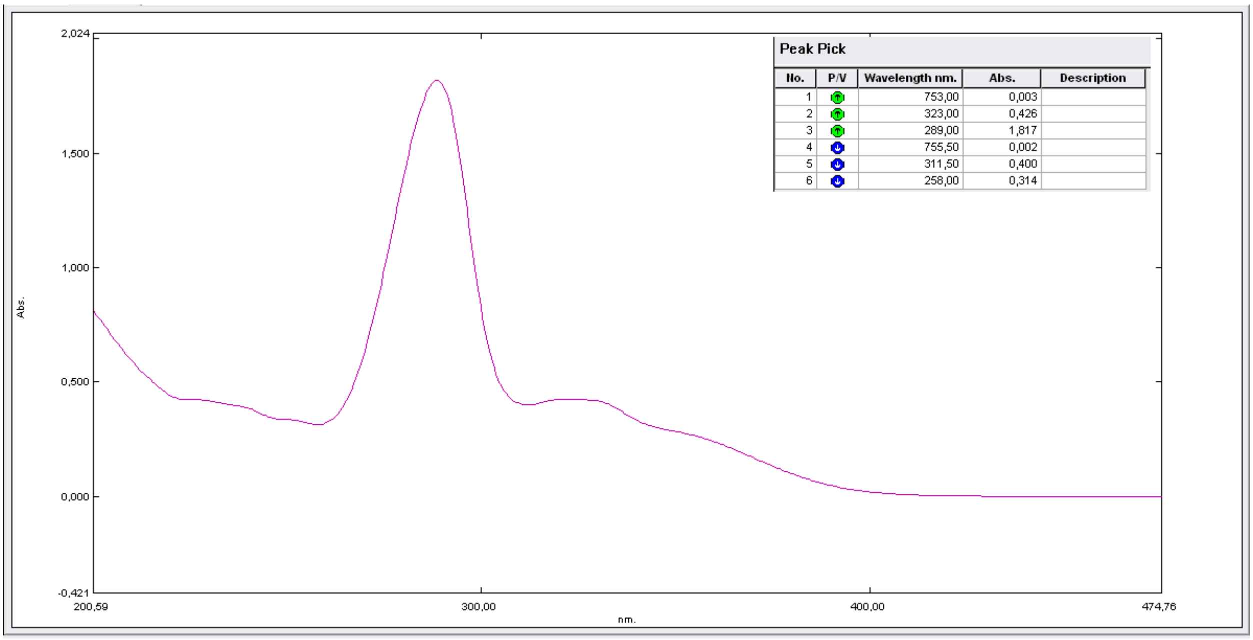

No.	P/V	Wavelength nm.	Abs.	Description
1		753,00	0,003	
2		323,00	0,426	
3		289,00	1,817	
4		755,50	0,002	
5		311,50	0,400	
6		258,00	0,314	

Figure S4. The UV spectrum of compound **7**.
