# Supplementary material for: Synthesis and Antimicrobial Activity of Novel Fluoroquinolone with Geranyl Amine Moiety
Source: Curr Issues Mol Biol. 2026 Feb 28;48(3):260. doi: 10.3390/cimb48030260 (PMC13024941; doi:10.3390/cimb48030260)

checkCIF/PLATON report

Structure factors have been supplied for datablock(s) dp334_0m

THIS REPORT IS FOR GUIDANCE ONLY. IF USED AS PART OF A REVIEW PROCEDURE FOR PUBLICATION, IT SHOULD NOT REPLACE THE EXPERTISE OF AN EXPERIENCED CRYSTALLOGRAPHIC REFEREE.

No syntax errors found. CIF dictionary Interpreting this report

Datablock: dp334_0m

```
Bond precision:   C-C = 0.0024 A          Wavelength=0.71073

Cell:            a=13.2222(13)     b=13.9846(13)      c=13.5854(13)
                 alpha=90          beta=118.074(3)     gamma=90
Temperature:     150 K

                 Calculated                  Reported
Volume           2216.5(4)                   2216.5(4)
Space group      P 21/c                      P 21/c
Hall group       -P 2ybc                     -P 2ybc
Moiety formula   C24 H29 F N2 O4             C24 H29 F N2 O4
Sum formula      C24 H29 F N2 O4             C24 H29 F N2 O4
Mr               428.49                      428.49
Dx,g cm-3        1.284                       1.284
Z                4                           4
Mu (mm-1)        0.093                       0.093
F000             912.0                       912.0
F000'            912.47
h,k,lmax         16,17,17                    16,17,17
Nref             4837                        4833
Tmin,Tmax        0.954,0.965                 0.559,0.746
Tmin'            0.954

Correction method= # Reported T Limits: Tmin=0.559 Tmax=0.746
AbsCorr = MULTI-SCAN
```

Data completeness= 0.999 Theta(max)= 26.998

R(reflections)= 0.0450(4044) wR2(reflections)= 0.1257(4833)

S = 1.034 Npar= 292

The following ALERTS were generated. Each ALERT has the format
test-name_ALERT_alert-type_alert-level.
Click on the hyperlinks for more details of the test.

Alert level C

```
PLAT220_ALERT_2_C NonSolvent   Resd 1  C   Ueq(max)/Ueq(min) Range        4.3 Ratio
PLAT222_ALERT_3_C NonSolvent Resd 1  H   Uiso(max)/Uiso(min) Range        4.9 Ratio
PLAT242_ALERT_2_C Low    'MainMol' Ueq as Compared to Neighbors of        C17 Check
PLAT355_ALERT_3_C Long   O-H (X0.82,N0.98A)  O3       - H3       .       1.01 Ang.
PLAT906_ALERT_3_C Large K Value in the Analysis of Variance ......      2.353 Check
PLAT911_ALERT_3_C Missing FCF Refl Between Thmin & STh/L=    0.600          3 Report
                3  2  0,  -3  0  2,  -3  3  2,
```

Alert level G

```
PLAT380_ALERT_4_G Incorrectly? Oriented X(sp2)-Methyl Moiety .....        C11 Check
PLAT380_ALERT_4_G Incorrectly? Oriented X(sp2)-Methyl Moiety .....        C21 Check
PLAT380_ALERT_4_G Incorrectly? Oriented X(sp2)-Methyl Moiety .....        C23 Check
PLAT910_ALERT_3_G Missing FCF Reflection(s) Below Theta(Min)[Deg]=       2.24 Note
                1  0  0,
PLAT913_ALERT_3_G Missing # of Very Strong Reflections in FCF ....          3 Note
                3  2  0,  -3  0  2,  -3  3  2,
PLAT933_ALERT_2_G Number of HKL-OMIT Records in Embedded .res File          1 Note
                1  0  0,
PLAT967_ALERT_5_G Note: Two-Theta Cutoff Value in Embedded .res ..       54.0 Degree
PLAT969_ALERT_5_G The 'Henn et al.' R-Factor-gap value ..........      2.730 Note
              Predicted wR2: Based on SigI**2  4.61 or SHELX Weight 12.16
PLAT978_ALERT_2_G Number C-C Bonds with Positive Residual Density.         14 Info
```

---

0 **ALERT level A** = Most likely a serious problem - resolve or explain
0 **ALERT level B** = A potentially serious problem, consider carefully
6 **ALERT level C** = Check. Ensure it is not caused by an omission or oversight
9 **ALERT level G** = General information/check it is not something unexpected

0 ALERT type 1 CIF construction/syntax error, inconsistent or missing data
4 ALERT type 2 Indicator that the structure model may be wrong or deficient
6 ALERT type 3 Indicator that the structure quality may be low
3 ALERT type 4 Improvement, methodology, query or suggestion
2 ALERT type 5 Informative message, check

It is advisable to attempt to resolve as many as possible of the alerts in all categories. Often the minor alerts point to easily fixed oversights, errors and omissions in your CIF or refinement strategy, so attention to these fine details can be worthwhile. It is up to the individual to critically assess their own results and, if necessary, seek expert advice.

PLATON version of 26/09/2025; check.def file version of 20/09/2025

---

duplicate check

No duplication found

Datablock dp334_0m - ellipsoid plot

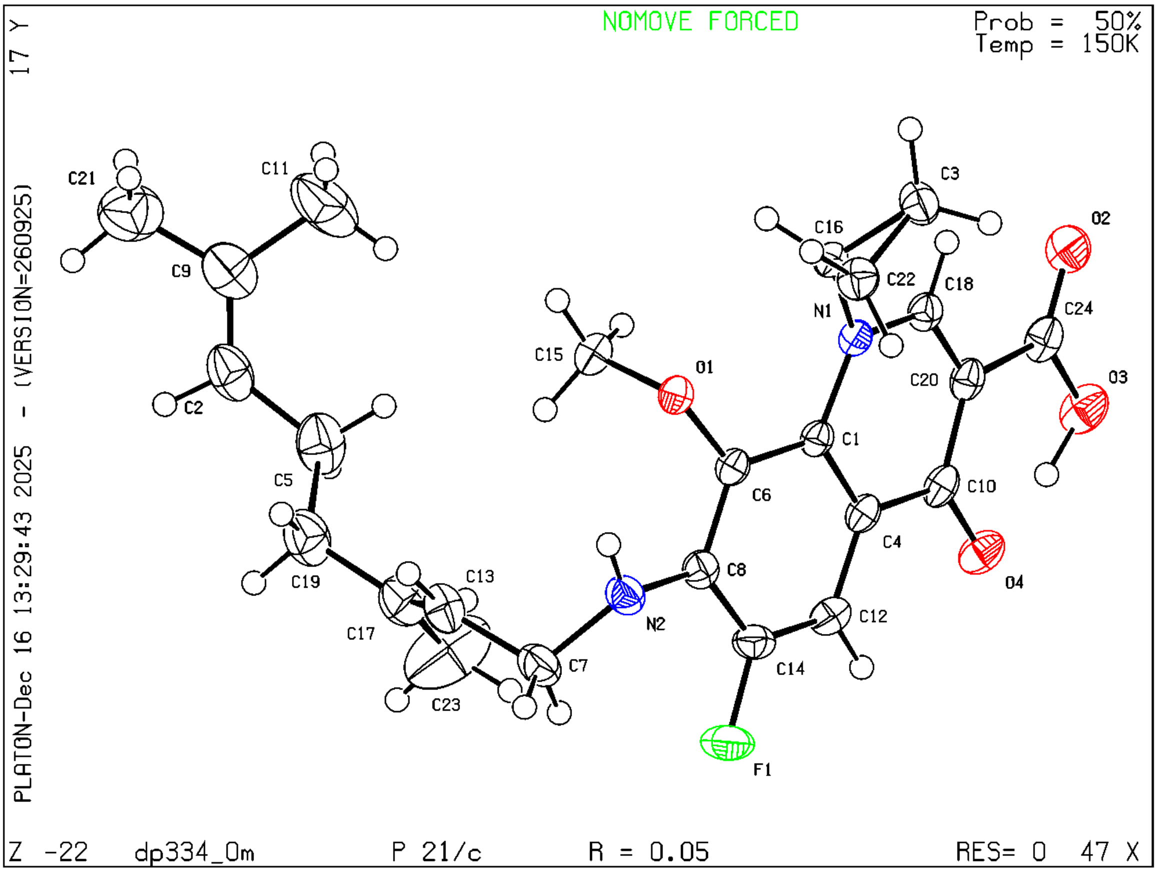

Supplement: Supplementary file 1 [file cimb-48-00260-s001.zip › Сheckcif.pdf]
