# Supplementary figures and images for: Synthesis and Antimicrobial Activity of Novel Fluoroquinolone with Geranyl Amine Moiety
Source: Curr Issues Mol Biol. 2026 Feb 28;48(3):260. doi: 10.3390/cimb48030260 (PMC13024941; doi:10.3390/cimb48030260)

### The IR spectrum of compound 7

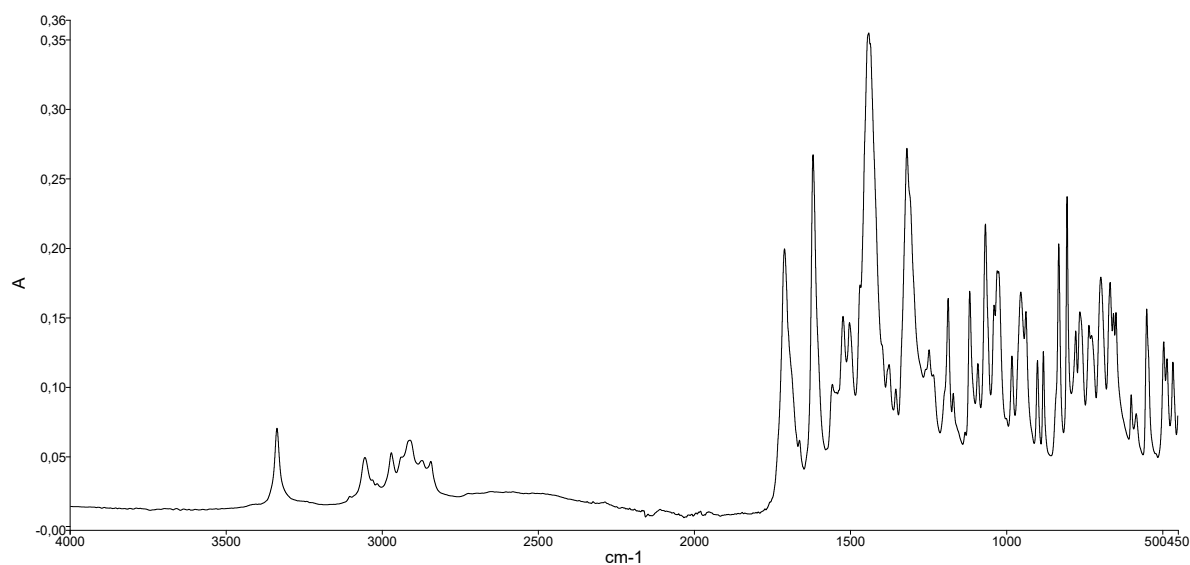

| Peak | X (cm <sup>-1</sup> ) | Y (A)  |
|------|-----------------------|--------|
| 1    | 3338,25               | 0,0705 |
| 2    | 3056,2                | 0,0494 |
| 3    | 2971,66               | 0,0529 |
| 4    | 2911,4                | 0,0619 |
| 5    | 1711,36               | 0,1999 |
| 6    | 1619,92               | 0,2677 |
| 7    | 1523,8                | 0,1512 |
| 8    | 1502,94               | 0,1467 |
| 9    | 1441,18               | 0,3555 |
| 10   | 1375,98               | 0,1164 |
| 11   | 1354,42               | 0,0988 |
| 12   | 1318,84               | 0,2724 |
| 13   | 1247,7                | 0,1272 |
| 14   | 1186,67               | 0,1641 |
| 15   | 1170,15               | 0,0956 |
| 16   | 1117,3                | 0,1693 |
| 17   | 1091,06               | 0,1171 |
| 18   | 1067,16               | 0,2178 |
| 19   | 1029,24               | 0,184  |
| 20   | 981,93                | 0,1225 |

|    |        |        |
|----|--------|--------|
| 21 | 953,68 | 0,1687 |
| 22 | 937,23 | 0,1546 |
| 23 | 900,17 | 0,1192 |
| 24 | 881,17 | 0,1258 |
| 25 | 831,81 | 0,2035 |
| 26 | 805,32 | 0,2374 |
| 27 | 777,33 | 0,1408 |
| 28 | 764,26 | 0,1545 |
| 29 | 735    | 0,1447 |
| 30 | 697,05 | 0,1795 |
| 31 | 667,23 | 0,1756 |
| 32 | 656,4  | 0,1533 |
| 33 | 648,41 | 0,154  |
| 34 | 599,65 | 0,0947 |
| 35 | 583,57 | 0,0811 |
| 36 | 549,82 | 0,1565 |
| 37 | 495,3  | 0,1329 |
| 38 | 484,86 | 0,1206 |
| 39 | 465,75 | 0,1181 |

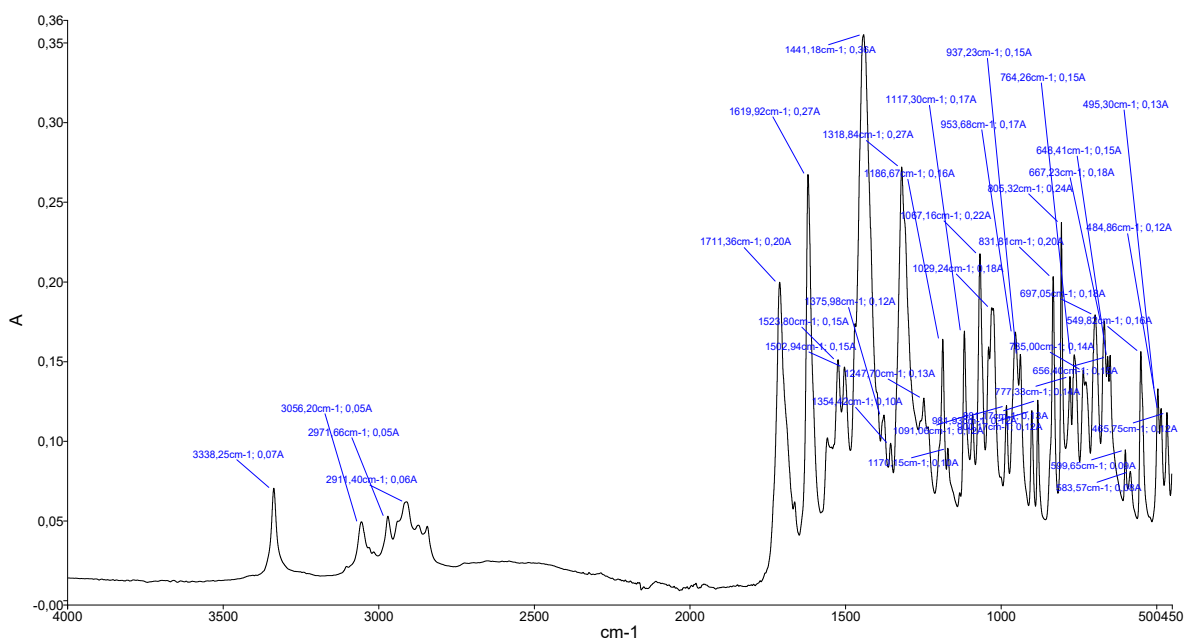

Supplement: Supplementary file 1 [file cimb-48-00260-s001.zip › IR spectrum.pdf]
